# Compact Acoustic Rainbow Trapping in a Bioinspired Spiral Array of Graded Locally Resonant Metamaterials

**DOI:** 10.3390/s19040788

**Published:** 2019-02-15

**Authors:** Liuxian Zhao, Shengxi Zhou

**Affiliations:** 1 Temasek Laboratories, Nanyang Technological University, 50 Nanyang Drive, Singapore 637553, Singapore; lzhao2@alumni.nd.edu; 2 School of Aeronautics, Northwestern Polytechnical University, Xi’an 710072, China

**Keywords:** acoustic rainbow trapping, artificial cochleae, Helmholtz resonator, spiral, locally resonant metamaterial

## Abstract

Acoustic rainbow trappers, based on frequency selective structures with graded geometries and/or properties, can filter mechanical waves spectrally and spatially to reduce noise and interference in receivers. These structures are especially useful as passive, always-on sensors in applications such as structural health monitoring. For devices that face space and weight constraints, such as microelectromechanical systems (MEMS) transducers and artificial cochleae, the rainbow trapping structures must be compact as well. To address this requirement, we investigated the frequency selection properties of a space-saving design consisting of Helmholtz resonators arranged at sub-wavelength intervals along a cochlear-inspired spiral tube. The height of the Helmholtz resonators was varied gradually, which induced bandgap formation at different frequencies along the length of the spiral tube. Numerical simulations and experimental measurements of acoustic wave propagation through the structure showed that frequencies in the range of 1–10 kHz were transmitted to different extents along the spiral tube. These rainbow trapping results were achieved with a footprint that was up to 70 times smaller than the previous structures operating at similar bandwidths, and the channels are 2.5 times of the previous structures operating at similar bandwidths.

## 1. Introduction

Frequency selective structures, which act as passive spectral filters for electromagnetic and mechanical waves, play an important role in many engineering applications. For instance, they are often employed to isolate desired frequencies in the multimodal Lamb wave to improve the accuracy of structural health monitoring of thin plates [1,2,3,4], and reduce noise and interference in radio frequency (RF) receivers in electronic and biomedical devices [5,6]. In recent years, it has been shown that the use of frequency selective structures with graded geometric structures or properties can be used to filter waves spectrally and spatially [7,8,9,10,11,12,13,14]. This technique has come to be known as rainbow trapping and was originally applied on electromagnetic waves to control optical delays and allow temporary storage of light [15,16,17,18,19]. However, it has been extended to acoustic waves as well [20].

In applications with space and/or weight constraints, such as microelectromechanical systems (MEMS) acoustic transducers [21,22,23] and artificial cochleae [24,25,26,27,28], the rainbow trapping structures need to be compact without altering the operational bandwidth. Meanwhile, a requirement that current studies continue to overlook is needed. For instance, in their proposed design for an artificial cochlear that realizes rainbow trapping of acoustic frequencies up to 10 kHz, Foucaud et al. [29] made use of a long (~1 m), straight plate of varying width, which was fabricated using traditional machining techniques that cannot be easily extended to produce miniature structures. On the other hand, White et al. [30] employed microfabrication techniques to manufacture a device, consisting of polyimide membranes and silicon nitride beams, which was less than 1 cm^3^ in volume. However, its operational frequency was extended to 35 kHz, which is too high for the use in an artificial human cochlear.

To resolve this issue, we propose an acoustic rainbow trapping design that was inspired by the coiled shape of the cochlear, which had evolved naturally from the need to perform spatial–spectral isolation of acoustic waves in a tight space [28,31,32]. Helmholtz resonators [33,34,35,36,37,38,39,40], a locally resonant metamaterial that can be positioned at subwavelength intervals to enhance the transmission loss of specific frequency bands [35,41,42,43], were arranged in this bioinspired spiral formation to further minimize the form factor of the design. The rainbow trapping function of the structure was realized by varying the height of the Helmholtz resonators, as shown in the numerical simulations and experiments in the following sections.

## 2. Numerical Simulation

### 2.1. Numerical Model

**Figure 1:**
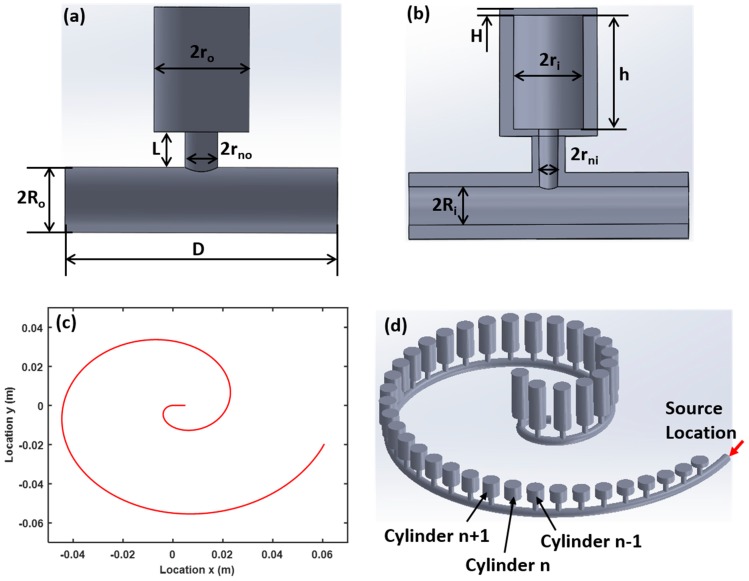
Schematic illustration of the cochlear-inspired structure. (**a**) Front view of Helmholtz resonator unit cell. (**b**) Cross-sectional view of the Helmholtz resonator unit cell showing the hollow interior of the cylinder and spiral tube. (**c**) Archimedean spiral and (**d**) isometric view of the entire structure.

In this study, we implement the rainbow trapping of acoustic waves using 40 individual Helmholtz resonators (Figure 1a,b) attached to a hollow tube in an Archimedean spiral configuration (Figure 1c), which can be described by the following equation using polar coordinates (*r*, *θ*): x=rθcos(θ) and y=rθsin(θ). In this paper, *r* = 20 mm, and *θ* changes from 0 to 2.1π. The total length of the spiral is *l* = 0.3 m, coiled within an area *l_a_* × *w_a_* = 0.1 m × 0.1 m. The periodical length of the Helmholtz resonators is *a* = 7 mm. All the Helmholtz resonators have the same cylinder inner radius *r_i_* = 2.5 mm, cylinder outer radius *r_o_* = 3.5 mm, cylinder top and bottom thickness *H* = 0.5 mm, neck inner radius *r_ni_* = 0.7 mm, neck outer radius *r_no_* = 1 mm, neck length *L* = 4 mm, duct inner radius *R_ni_* = 1 mm and duct outer radius *R_no_* = 1.5 mm. The cylinder height, *h*, is increased in discrete steps of *δ* = 0.359 mm from 1 mm (for the first cylinder (*n* = 1)) to 15 mm for the last cylinder (*n* = 40) (Figure 1d).

For the dispersion curve analysis, the structure is assumed to be infinite and periodic in the direction of wave propagation (*x*) with the period *a* and. According to the Floquet–Bloch theorem, the relation for the pressure distribution (*p*) can be expressed as [44]:

(1) p(x+a)=p(x)exp[i(ka)]

where k is the wavenumber of the acoustic wave.

For the spatial-spectral analysis, the equation used to analyze the acoustic wave problems is expressed as [44]:

(2) $$\nabla \cdot \left(-\frac{1}{\rho }\nabla p\right)-\frac{\omega^{2}p}{\rho c^{2}}=0$$

where $p=p_{0}e^{i\omega t}$, the input pressure amplitude is $p_{0}=1\;Pa,$ ρ is the density of air ($\rho =1.225\;\mathrm{kg}/m^{3}$), *c* is the acoustic wave speed in air (*c* = 343 m/s).

For the element size used in these studies, we choose the “physics-controlled mesh” type with “finer” element size.

### 2.2. Dispersion Analysis

Since the cylinder height is designed to increase linearly along the length of the duct for the graded Helmholtz resonators and the increment *δ* = 0.359 mm between two consecutive cylinders are reasonably small, we can consider the *n*-th cylinder with the height *h_n_* in the graded Helmholtz resonators as a cylinder with the same height *h_n_* in a series of the periodic Helmholtz resonators. Therefore, we could use the dispersion characteristics of the periodic Helmholtz resonators to approximate those of the *n*-th cylinder in a set of the graded Helmholtz resonators [20].

**Figure 2:**
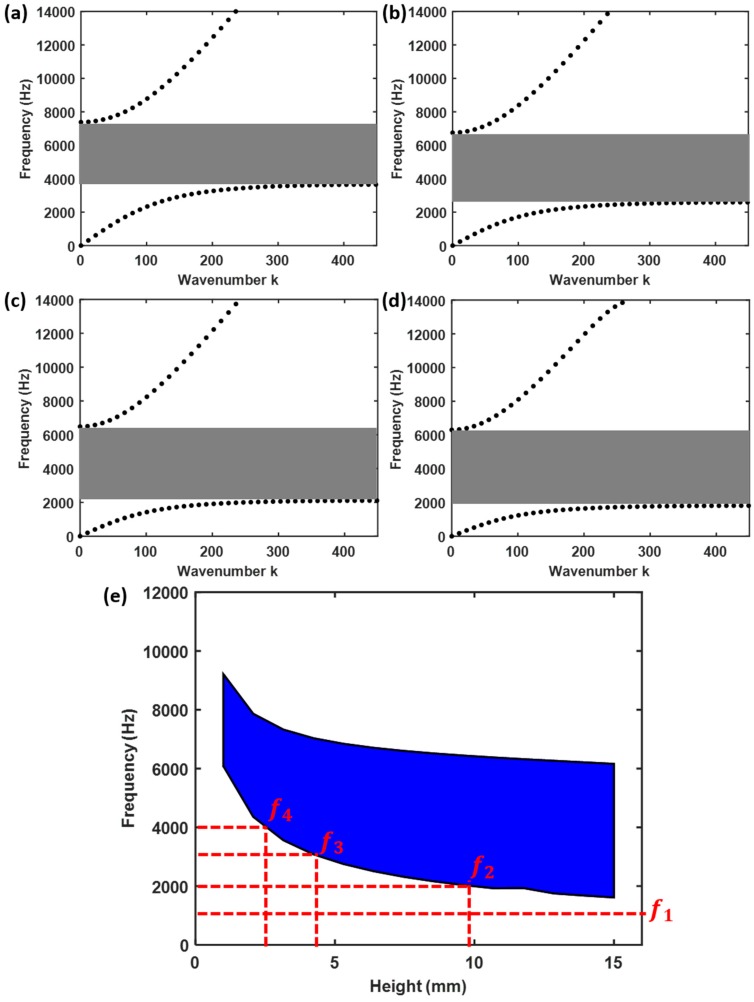
Dispersion curve analysis of graded Helmholtz resonators. Frequency-wavenumber dispersion curves in the first Brillouin zone for unit cell with cylinder height (**a**) h = 3 mm, (**b**) h = 6 mm, (**c**) h = 9 mm, (**d**) h = 12 mm. (**e**) Variation of bandgap with respect to cylinder height. The blue shaded area represents the bandgaps.

Since the bandgap of a Helmholtz resonator is strongly dependent on its geometry, the cylinders at different locations have different dispersion curves. To demonstrate the spatial evolution of dispersion curves in the graded Helmholtz resonators, Figure 2a–d presents the frequency-wavenumber dispersion curves in the first Brillouin zone for cylinders with different heights: *h*_7_ = 3.1 mm (the 7th cylinder), *h*_15_ = 6.0 mm (the 15th cylinder), *h*_24_ = 9.2 mm (the 24th cylinder), *h*_32_ = 12.1 mm (the 32nd cylinder). From Figure 2a–d, we can see there is a bandgap for each Helmholtz resonator, for which acoustic waves in those frequencies are not allowed to propagate.

Figure 2e shows the frequency variation of acoustic wave bandgap (shaded in blue) with respect to cylinder height in the graded Helmholtz resonators. With an increase of cylinder height, the width of the bandgap becomes larger, and the bandgap gradually shifts to lower frequencies. Hence, if acoustic waves that carry frequencies in the range of 1614 Hz–6083 Hz transmit the graded Helmholtz resonators from cylinders *n* = 1 to *n* = 40, the different frequency components will propagate to different extents. For instance, *f*_1_ = 1 kHz will be able to propagate to all the Helmholtz resonators, while *f*_2_ = 2 kHz can only propagate to resonators with *h* < 10.69 mm. Similarly, *f*_3_ = 3 kHz and *f*_4_ = 4 kHz can only propagate to resonators with *h* < 4.95 mm and *h* < 2.79 mm respectively (Figure 2e). In other words, the different frequencies in an acoustic wave will be spatially filtered into 1 of the 40 Helmholtz resonators, each acting as a spatial–spectral channel.

### 2.3. Spatial–Spectral Analysis

To ascertain the above expectation that different frequencies propagate to different extents in the spiral structure, finite element simulations are performed using COMSOL Multiphysics 5.3 [45]. An incident pressure field is applied for excitation starting from *n* = 1 using four different frequencies *f*_1_ = 1 kHz, *f*_2_ = 2 kHz, *f*_3_ = 3 kHz and *f*_4_ = 4 kHz.

**Figure 3:**
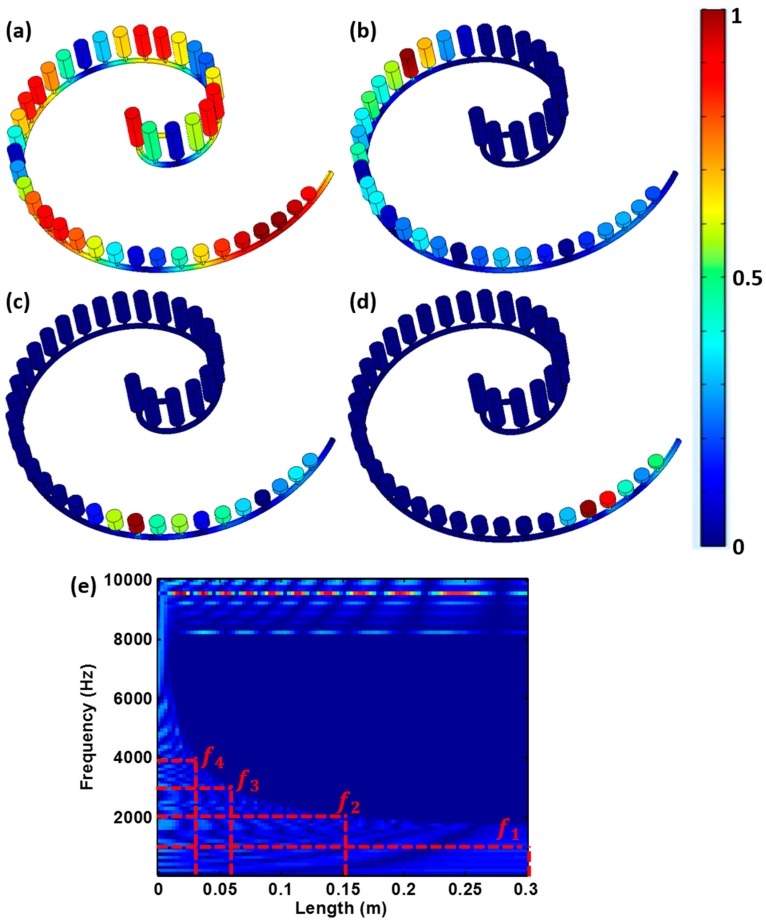
Normalized acoustic pressure in the spiral structure with Helmholtz resonators for (**a**) *f*_1_ = 1 kHz, (**b**) *f*_2_ = 2 kHz, (**c**) *f*_3_ = 3 kHz, (**d**) *f*_4_ = 4 kHz, and (**e**) normalized pressure amplitude (brighter spots indicate higher values) for each frequency along the central axis of the spiral tube.

Results presented in Figure 3a–d indicate that the acoustic wave can propagate through all the graded Helmholtz resonators at the frequency *f*_1_ = 1 kHz, which is not within the bandgap of any cylinder. In contrast, the acoustic wave would not propagate beyond the 28-th, 12-th, and 6-th cylinders for the frequencies *f*_2_ = 2 kHz, *f*_3_ = 3 kHz and *f*_4_ = 4 kHz, respectively. These results are markedly different from those obtained when the graded Helmholtz resonators are conjoined together graded [46]. In such structures, each resonator can only support a narrow range of frequencies and the tight mechanical coupling between adjacent resonators prevents complete rainbow trapping from being achieved i.e. waves of a single frequency will be “trapped” at the target resonator, as well as its neighbors.

A more comprehensive examination is conducted using a frequency sweep, *f* = 1 kHz–10 kHz, of the excitation source, which contains a broadband frequency information. The excitation signal used in this study is similar to the broadband pulse used in [47]. The frequency-space representation of normalized pressure amplitude, distributed along the Archimedean spiral shape, was plotted in Figure 3e. It clearly shows that acoustic waves of different frequencies stopped propagating forward and concentrated their wave energy at different locations along the spiral tube. The height of the Helmholtz resonators and the “trapped” wave frequencies at each of these locations correspond to the lower boundary of the bandgap as shown in Figure 2e. In addition, it is observed that with increasing excitation frequency, the wave propagation distance along the spiral tube became shorter and the location with concentrated energy gradually shifted towards the source. Note that the upper frequency range (> 8kHz) means that the signals can propagate through the entire structure, that’s why the pressure are very high over the entire length range. This result is consistent with the bandgap plot in Figure 2e, because when the frequency over 8 kHz, it is a passband.

**Figure 4:**
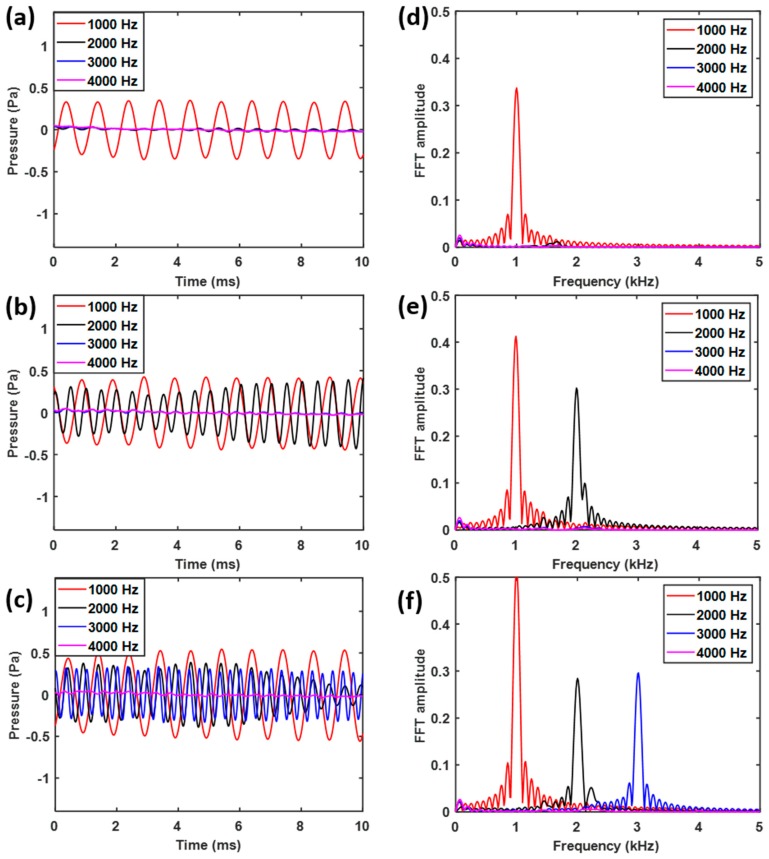
Pressure level at the (**a**) 37th (**b**) 17th and (**c**) 7th cylinders, and the corresponding Fourier spectrum of the pressure level at the (**d**) 37th (**e**) 17th and (**f**) 7th cylinders.

These observations are supported by the time dependent pressure wave results obtained from the 37th (Figure 4a), 17th (Figure 4b) and 7th (Figure 4c) cylinders, as well as their Fast Fourier transform (FFT) (Figure 4d–f). This clearly shows that only acoustic waves with the frequency of *f*_1_ = 1 kHz can reach the 37th cylinder, while waves with the frequencies *f*_1_ = 1 kHz and *f*_2_ = 2 kHz were present in the 17th cylinder, and the frequencies *f*_1_ = 1 kHz, *f*_2_ = 2 kHz and *f*_3_ = 3 kHz were found in the 7th cylinder. These results are consistent with the implications derived from the bandgap plot in Figure 2e and demonstrate the rainbow trapping potential of the spiral structure.

## 3. Experiments

### 3.1. Experimental Setup

**Figure 5:**
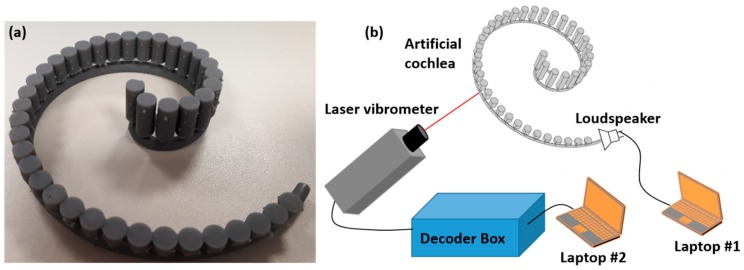
Experimental verification of artificial cochlea for acoustic wave trapping, (**a**) 3D printed spiral structure with Helmholtz resonators, (**b**) schematic of the experimental setup. A loudspeaker is connected to the opening of the spiral tube to generate acoustic waves. A point laser Doppler vibrometer was used to measure the acoustic waves at three different locations.

A proof-of-concept experiment is conducted on a sample with the same dimensions as the numerical model described in Figure 1. The sample is 3D printed with the Grey Resin, which has a high stiffness after curing [48], using a commercial stereolithography 3D printer, Form 2 (Formlabs Inc., USA) (Figure 5a). A single hole with diameter of 1 mm is drilled in the hollow tube at the location of the 7th, 17th and 37th cylinders and covered over with reflective tapes for acoustic wave measurements using a point laser Doppler vibrometer (Polytech GmbH, Germany). The scanning laser vibrometer is used to measure the particle velocity on the reflective tapes. The measured particle velocity amplitude on the reflective tapes is proportional to the pressure of sound waves, which is used to reflect the sound wave amplitude at different frequency ranges. The vibrometer is connected to a decoder box for acquiring the particle velocity. A small loudspeaker is used to generate the acoustic wave at different frequencies. In this study, there are four tests for each hole at the frequencies of *f*_1_ = 1 kHz, *f*_2_ = 2 kHz, *f*_3_ = 3 kHz and *f*_4_ = 4 kHz.

### 3.2. Experimental Results

**Figure 6:**
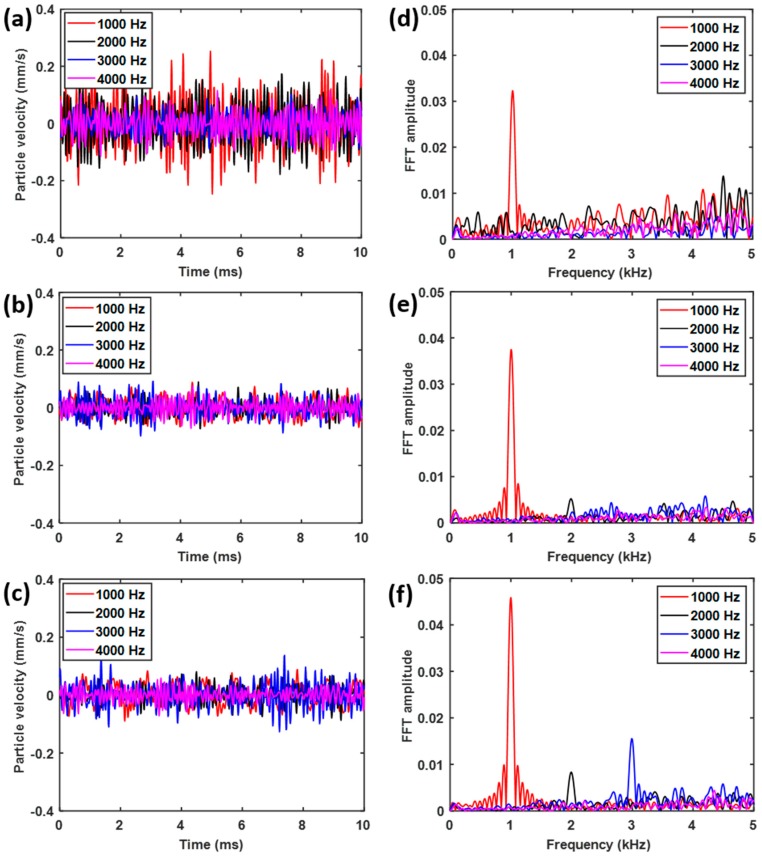
Particle velocities, which are proportional to the local pressure, in the temporal domain and their frequency spectrum, (**a**)–(**c**) particle velocities at the hollow tube corresponding to locations of the 37th, 17th and 7th cylinders, and (**d**)–(**f**) their Fourier spectrum correspondingly.

The experimental results are shown in Figure 6. They are very similar to those predicted in Figure 4, except that shorter peaks are observed for *f*_2_ = 2 kHz (Figure 6e,f) and *f*_3_ = 3 kHz (Figure 6f). This is likely a combined result of attenuation along the spiral tube and background noise. The background noise is due to the broadband white noise exists everywhere and this experiment is performed without echoless chamber. Nevertheless, Figure 6 clearly indicates that the spiral structure with the Helmholtz resonators does indeed possess rainbow trapping capabilities, in accordance to the results obtained from numerical simulations. Moreover, this spatial-spectral filtering of acoustic waves is realized in a structure with a footprint up to 70 times smaller [29], and with the channels as many as 1.3 to 2.5 times [20,49] of that of previous designs operated in similar frequency ranges. Further optimization of the current design is expected to lead to even higher channel densities for acoustic rainbow trapping.

## 4. Conclusions

We have numerically and experimentally demonstrated that broadband acoustic waves can be filtered spectrally at different spatial locations when propagating along a spiral array of the Helmholtz resonators, which have subwavelength periods and a graded height. The linear variation in height led to a systematic modulation of the acoustic bandgap along the spiral, resulting in the rainbow trapping effect. This was verified through numerical simulations and experiments. The results showed that a total length of *l* = 0.3 m of the graded frequency selective structure with 40 spatial-spectral channels can be fabricated within an area of *l_a_* × *w_a_* = 0.1 m × 0.1 m, which was up to 70 times smaller and had up to 2.5 times as many channels as that of previous structures operating in similar bandwidths.

## References

[1] Suhaimi S.A., Azemi S.N., Jack S.P. Structural health monitoring system using 3d frequency selective surface. Proceedings of the 2016 IEEE Asia-Pacific Conference on Applied Electromagnetics (APACE).

[2] Sang-Dong J., Byung-Woo K., Jaehwan K. (2013). Frequency selective surface based passive wireless sensor for structural health monitoring. Smart Mater. Struct..

[3] Sung G.H., Sowerby K.W., Neve M.J., Williamson A.G. (2006). A frequency-selective wall for interference reduction in wireless indoor environments. IEEE Antennas Propag. Mag..

[4] Zhao L., Yang J., Wang K.W., Semperlotti F. (2015). An application of impediography to the high sensitivity and high resolution identification of structural damage. Smart Mater. Struct..

[5] Zhao J., Zhang Q., Wang R., Chen Y. Incident-Insensitive Frequency Selective Surface using degradable material for bio-medical application. Proceedings of the 2014 Asia-Pacific Microwave Conference.

[6] Seman F.C., Cahill R., Fusco V.F., Goussetis G. (2011). Design of a salisbury screen absorber using frequency selective surfaces to improve bandwidth and angular stability performance. IET Microw. Antennas Propag..

[7] Tsakmakidis K.L., Boardman A.D., Hess O. (2007). ‘Trapped rainbow’ storage of light in metamaterials. Nature.

[8] Gan Q., Ding Y.J., Bartoli F.J. (2009). “rainbow’’ trapping and releasing at telecommunication wavelengths. Phys. Rev. Lett..

[9] Williams C.R., Andrews S.R., Maier S.A., Fernández-Domínguez A.I., Martín-Moreno L., García-Vidal F.J. (2008). Highly confined guiding of terahertz surface plasmon polaritons on structured metal surfaces. Nat. Photonics.

[10] Gan Q., Gao Y., Wagner K., Vezenov D., Ding Y.J., Bartoli F.J. (2011). Experimental verification of the rainbow trapping effect in adiabatic plasmonic gratings. Proc. Natl. Acad. Sci. USA.

[11] Jang M.S., Atwater H. (2011). Plasmonic rainbow trapping structures for light localization and spectrum splitting. Phys. Rev. Lett..

[12] Kats M.A., Woolf D., Blanchard R., Yu N., Capasso F. (2011). Spoof plasmon analogue of metal-insulator-metal waveguides. Opt. Express.

[13] Chen L., Wang G.P., Gan Q., Bartoli F.J. (2009). Trapping of surface-plasmon polaritons in a graded bragg structure: Frequency-dependent spatially separated localization of the visible spectrum modes. Phys. Rev. B.

[14] He S., He Y., Jin Y. (2012). Revealing the truth about ‘trapped rainbow’ storage of light in metamaterials. Sci. Rep..

[15] Noda S., Chutinan A., Imada M. (2000). Trapping and emission of photons by a single defect in a photonic bandgap structure. Nature.

[16] Julsgaard B., Sherson J., Cirac J.I., Fiurášek J., Polzik E.S. (2004). Experimental demonstration of quantum memory for light. Nature.

[17] Xia F., Sekaric L., Vlasov Y. (2006). Ultracompact optical buffers on a silicon chip. Nat. Photonics.

[18] Yanik M.F., Fan S. (2007). Dynamic photon storage. Nat. Phys..

[19] Fiore V., Yang Y., Kuzyk M.C., Barbour R., Tian L., Wang H. (2011). Storing optical information as a mechanical excitation in a silica optomechanical resonator. Phys. Rev. Lett..

[20] Zhu J., Chen Y., Zhu X., Garcia-Vidal F.J., Yin X., Zhang W., Zhang X. (2013). Acoustic rainbow trapping. Sci. Rep..

[21] Senesi M., Ruzzene M. (2011). A frequency selective acoustic transducer for directional lamb wave sensing. J. Acoust. Soc. Am..

[22] Baravelli E., Senesi M., Ruzzene M., Marchi L.D., Speciale N. (2011). Double-channel, frequency-steered acoustic transducer with 2-d imaging capabilities. IEEE Trans. Ultrason. Ferroelectr. Freq. Control.

[23] Kusano Y., Segovia-Fernandez J., Sonmezoglu S., Amirtharajah R., Horsley D.A. Frequency selective mems microphone based on a bioinspired spiral-shaped acoustic resonator. Proceedings of the 2017 19th International Conference on Solid-State Sensors, Actuators and Microsystems (TRANSDUCERS).

[24] Tanaka K., Abe M., Ando S. (1998). A novel mechanical cochlea “fishbone” with dual sensor/actuator characteristics. IEEE/ASME Trans. Mechatron..

[25] Xu T., Bachman M., Zeng F.-G., Li G.-P. (2004). Polymeric micro-cantilever array for auditory front-end processing. Sens. Actuators A Phys..

[26] Chen F., Cohen H.I., Bifano T.G., Castle J., Fortin J., Kapusta C., Mountain D.C., Zosuls A., Hubbard A.E. (2006). A hydromechanical biomimetic cochlea: Experiments and models. J. Acoust. Soc. Am..

[27] Wittbrodt M.J., Steele C.R., Puria S. (2006). Developing a physical model of the human cochlea using microfabrication methods. Audiol. Neurotol..

[28] Shintaku H., Nakagawa T., Kitagawa D., Tanujaya H., Kawano S., Ito J. (2010). Development of piezoelectric acoustic sensor with frequency selectivity for artificial cochlea. Sens. Actuators A Phys..

[29] Foucaud S., Michon G., Gourinat Y., Pelat A., Gautier F. (2014). Artificial cochlea and acoustic black hole travelling waves observation: Model and experimental results. J. Sound Vib..

[30] White R.D., Grosh K. (2005). Microengineered hydromechanical cochlear model. Proc. Natl. Acad. Sci. USA.

[31] Atturo F., Schart-Morén N., Larsson S., Rask-Andersen H., Li H. (2018). The human cochlear aqueduct and accessory canals: A micro-ct analysis using a 3d reconstruction paradigm. Otol. Neurotol..

[32] Pietsch M., Aguirre Dávila L., Erfurt P., Avci E., Lenarz T., Kral A. (2017). Spiral form of the human cochlea results from spatial constraints. Sci. Rep..

[33] Kwon B.J., Jo C., Park K.C., Oh I.-K. (2013). Wave propagation characteristics of acoustic metamaterials with helmholtz resonators. Korean Sci..

[34] Liu B., Yang L. (2017). Transmission of low-frequency acoustic waves in seawater piping systems with periodical and adjustable helmholtz resonator. J. Mar. Sci. Eng..

[35] Yamamoto T. (2018). Acoustic metamaterial plate embedded with helmholtz resonators for extraordinary sound transmission loss. J. Appl. Phys..

[36] Yang X., Yin J., Yu G., Peng L., Wang N. (2015). Acoustic superlens using helmholtz-resonator-based metamaterials. Appl. Phys. Lett..

[37] Yu X., Lu Z., Cui F., Cheng L., Cui Y. (2017). Tunable acoustic metamaterial with an array of resonators actuated by dielectric elastomer. Extreme Mech. Lett..

[38] Casarini C., Windmill J.F.C., Jackson J.C. 3D printed small-scale acoustic metamaterials based on helmholtz resonators with tuned overtones. Proceedings of the 2017 IEEE SENSORS.

[39] Dubois M., Shi C., Wang Y., Zhang X. (2017). A thin and conformal metasurface for illusion acoustics of rapidly changing profiles. Appl. Phys. Lett..

[40] Faure C., Richoux O., Félix S., Pagneux V. (2016). Experiments on metasurface carpet cloaking for audible acoustics. Appl. Phys. Lett..

[41] Groby J.P., Lagarrigue C., Brouard B., Dazel O., Tournat V., Nennig B. (2015). Enhancing the absorption properties of acoustic porous plates by periodically embedding helmholtz resonators. J. Acoust. Soc. Am..

[42] Jiménez N., Huang W., Romero-García V., Pagneux V., Groby J.P. (2016). Ultra-thin metamaterial for perfect and quasi-omnidirectional sound absorption. Appl. Phys. Lett..

[43] Fang N., Xi D., Xu J., Ambati M., Srituravanich W., Sun C., Zhang X. (2006). Ultrasonic metamaterials with negative modulus. Nat. Mater..

[44] Elford D.P., Chalmers L., Kusmartsev F.V., Swallowe G.M. (2011). Matryoshka locally resonant sonic crystal. J. Acoust. Soc. Am..

[45] Comsol Multiphysics Version 5.3-Structural and Acoustics Module.

[46] Jiménez N., Romero-García V., Pagneux V., Groby J.-P. (2017). Rainbow-trapping absorbers: Broadband, perfect and asymmetric sound absorption by subwavelength panels for transmission problems. Sci. Rep..

[47] Vanhille C. (2017). Two-dimensional numerical simulations of ultrasound in liquids with gas bubble agglomerates: Examples of bubbly-liquid-type acoustic metamaterials (blamms). Sensors.

[48] Decker C. (2003). Kinetic study and new applications of uv radiation curing. Macromol. Rapid Commun..

[49] Ni X., Wu Y., Chen Z.-G., Zheng L.-Y., Xu Y.-L., Nayar P., Liu X.-P., Lu M.-H., Chen Y.-F. (2014). Acoustic rainbow trapping by coiling up space. Sci. Rep..

